# Potential for Inhalation Exposure to Engineered Nanoparticles from Nanotechnology-Based Cosmetic Powders

**DOI:** 10.1289/ehp.1104350

**Published:** 2012-03-06

**Authors:** Yevgen Nazarenko, Huajun Zhen, Taewon Han, Paul J. Lioy, Gediminas Mainelis

**Affiliations:** 1 Department of Environmental Sciences, Rutgers University, the State University of New Jersey, New Brunswick, New Jersey, USA; 2 Robert Wood Johnson Medical School–University of Medicine and Dentistry of New Jersey, Piscataway, New Jersey, USA; 3 Environmental and Occupational Health Sciences Institute, Robert Wood Johnson Medical School–University of Medicine and Dentistry of New Jersey and Rutgers University, Piscataway, New Jersey, USA

**Keywords:** consumer products, cosmetics, emerging contaminants, inhalation exposure, nanoaerosol, nanomaterials, nanoparticles, personal exposure

## Abstract

Background: The market of nanotechnology-based consumer products is rapidly expanding, and the lack of scientific evidence describing the accompanying exposure and health risks stalls the discussion regarding its guidance and regulation.

Objectives: We investigated the potential for human contact and inhalation exposure to nanomaterials when using nanotechnology-based cosmetic powders and compare them with analogous products not marketed as nanotechnology based.

Methods: We characterized the products using transmission electron microscopy (TEM) and laser diffraction spectroscopy and found nanoparticles in five of six tested products. TEM photomicrographs showed highly agglomerated states of nanoparticles in the products. We realistically simulated the use of cosmetic powders by applying them to the face of a human mannequin head while simultaneously sampling the released airborne particles through the ports installed in the mannequin’s nostrils.

Results: We found that a user would be exposed to nanomaterial predominantly through nanoparticle-containing agglomerates larger than the 1–100-nm aerosol fraction.

Conclusions: Predominant deposition of nanomaterial(s) will occur in the tracheobronchial and head airways—not in the alveolar region as would be expected based on the size of primary nanoparticles. This could potentially lead to different health effects than expected based on the current understanding of nanoparticle behavior and toxicology studies for the alveolar region.

The development of nanotechnologies leads to the incorporation of nanomaterials into common consumer products because of the novelty and distinctive properties of materials at nanoscale. The number of nanotechnology-based consumer products listed in the Nanotechnology Consumer Products Inventory (Woodrow Wilson International Center for Scholars 2011a) is currently > 1,300. These products are manufactured by nearly 600 companies in 30 countries. Because this online database lists only a subset of products advertised on the Internet as nanotechnology based (Woodrow Wilson International Center for Scholars 2011b), the actual number is probably higher. The expansion of the nanotechnology-based consumer products market (Bradford et al. 2009; Kessler 2011; Maynard 2007; Paull and Lyons 2008; Woodrow Wilson International Center for Scholars 2011b) is cause for concern regarding potential human exposure to nanomaterials and possible health risks. The potential for exposure is still poorly understood, and potential health effects are unknown (Drobne 2007; Frater et al. 2006; Segal 2004; Van Calster 2006; Warheit et al. 2007). This impedes the development of appropriate consumer safety regulations and guidelines (Maynard et al. 2006; Oberdörster et al. 2005a; Paull and Lyons 2008; Riediker 2009).

A nanoproduct’s type and intended use determine the most plausible routes and extent of exposure (Oberdörster et al. 2005b; Wardak et al. 2008). Use of nanotechnology-based cosmetic powders and sprays could lead to especially high levels of dermal and inhalation exposure, the latter being a consequence of product application leading to aerosol generation in the personal breathing zone (Hansen et al. 2008; Shimada et al. 2009). Contradictory conclusions regarding dermal absorption and toxicity of nanoparticles have been reported (Baroli 2009; Baroli et al. 2007; Crosera et al. 2009; Larese et al. 2009; Senzui et al. 2010), and additional research has been recommended to better characterize and determine health concerns associated with dermal nanomaterial exposure (Crosera et al. 2009). At the same time, inhalation exposure to nanomaterials is a serious health concern (Savolainen et al. 2010). During consumer use, nanomaterials can be released and enter the respiratory system as free nanoparticles, nanoparticle agglomerates, and nanoparticles within or attached to larger particles. Additionally, other substances present in the applied nanoproduct could be physically transported on the nanoparticles themselves (Nowack and Bucheli 2007).

Many studies investigating the toxicity of pure nanomaterials have already been performed and summarized (Holgate 2010; Johnston et al. 2010; Marambio-Jones and Hoek 2010; Ostrowski et al. 2009; Savolainen et al. 2010; Schilling et al. 2010). However, the potential for consumer exposure to nanoparticles from actual nanotechnology-based products where nanomaterials exist in a product matrix with other ingredients has so far been addressed to only a limited degree.

Potential exposures and associated health effects are expected to depend on the dispersed particle size, agglomeration state, surface area and chemistry, solubility, concentration, and possibly the shape characteristics of nanomaterial(s) in a product (Bermudez et al. 2004; Shrader-Frechette 2007). Initial nanomaterial ingredients in consumer products might be chemically and physically modified through interactions with other ingredients in the product or through nanoparticle surface treatment during production, which may also affect their toxicity (Kessler 2011; Warheit et al. 2005). Therefore, properties of original nanomaterial ingredients cannot serve as the sole basis for predicting exposure and health effects of a particular nanotechnology-based consumer product (Lioy et al. 2010; Maynard 2007). The size distribution of aerosol particles released and potentially inhaled during product use may also depend on the composition of the product, which in turn would affect the deposition of nanomaterial(s) in the respiratory system. Thus, one should characterize not only the in-product nanomaterials but also their characteristics during actual use by simulation and investigation of realistic exposure scenarios (Lioy 2010; Nazarenko et al. 2011).

In our earlier research, we investigated nanotechnology-based consumer spray products as well as their regular, non–nanotechnology-based, counterparts in a realistic exposure scenario, including a simulated application of the sprays (Nazarenko et al. 2011). The study demonstrated the potential for inhalation exposure to nanosize particles from all investigated products. Release of airborne silver nanoparticles during propellant-facilitated spraying of one nanotechnology-based silver spray was also shown in another study (Hagendorfer et al. 2010). The magnitude and prevalence of such exposures and associated risks are still unknown (Bradford et al. 2009; Keenan et al. 2009; Lioy et al. 2010).

In this study we focused on cosmetic powders, including nanotechnology-based- and non–nanotechnology-based powders, another category of consumer products with a high probability of inhalation exposure. The study had the following objectives: *a*) to characterize nanoparticles in several nanotechnology-based cosmetic powders currently in the market, *b*) to determine the potential for exposures to airborne nanoparticles and their agglomerates during the use of cosmetic powders in a realistic exposure scenario, and *c*) to compare investigated nanotechnology-based cosmetic powders with their regular (non-nanotechnology) counterparts. This study responds to the call for independent nanotechnology-based commercial consumer product research, free of potential conflict of interest (Maynard 2007; Maynard and Aitken 2007; Michelson 2008; Shrader-Frechette 2007; Thomas et al. 2009).

To the best of our knowledge, this study is the first to determine the potential for human inhalation exposure to nanomaterials released from nanotechnology-based and regular cosmetic powders in a realistic exposure simulation.

## Materials and Methods

*Tested cosmetic powders.* We selected three nanotechnology-based cosmetic powders (“nanopowders”)—a moisturizer, a blusher, and a loose powder sunscreen—from the Woodrow Wilson Nanotechnology Consumer Products Inventory (Woodrow Wilson International Center for Scholars 2011a) and acquired them from the manufacturers. Currently, reporting by the manufacturers is the only way to identify the “nano” status of the products, and it may not be a guarantee that any given product in the inventory contains nanotechnological components (Hansen et al. 2008; Som et al. 2010). Consequently, the authors’ references to products in this project as “nanoproducts” or “nanopowders” are based on product’s presence in the above-mentioned inventory as of 1 September 2008. Additionally, we selected three cosmetic powders that manufacturers do not claim include nanomaterial(s) (“regular powders”)—two blot powders and a finishing powder—and tested them for comparison with the cosmetic nanopowders. The selected regular powders perform functions similar to those of their nanotechnology-based counterparts and are also applied to the face.

The studied nano- and regular products are listed in Supplemental Material, Table 1 (http://dx.doi.org/10.1289/ehp.1104350), along with their intended application purpose and composition as reported by the manufacturers. We tested all products in their original formulation as shipped, without any deliberate pretreatment, deagglomeration, or any other type of modification. We replaced the product brand names with letter codes.

**Table 1:** Characteristics of the tested cosmetic powder products obtained using different analysis methods.

LDS diameter			Mannequin sampling mode diameter		
Smallest detected particle (nm)	Mode (μm)				
		Product	Summary of TEM results^a^	Presence of particles < 100 nm	SMPS (nm)	APS (μm)
Nanopowdersb												
M		6–45 nm, only agglomerates, fused spheroidal and irregular, solid, beam insensitive		All particles are < 100 nm and agglomerated		100		0.33		< 100		1.7
D		> 5 μm, single particles, irregular, solid, beam insensitive		Not observed		440		0.66		< 100		1.0
K		7 nm to > 3 μm, only agglomerates, angular spheroidal, solid, beam insensitive		Many agglomerated particles < 100 nm		100		0.33		53.3, 101.8, 241.4, 358.7		1.5
Regular powders												
F		12 nm to > 8.8 μm, single particles and agglomerates, angular composite, beam insensitive		Many particles < 100 nm, but all in composites within large particles		100		0.33		< 100 nm, 121.9		2.6
G		62.5 nm to > 10 μm, single particles and agglomerates, irregular, solid, beam insensitive		Very few separate particles, unclear if larger particles are agglomerates of nanoparticles		100		0.33		156.8		2.6
E		23.3 nm to > 12.8 μm, single particles and agglomerates, spheroidal, solid, beam insensitive		Many agglomerated and attached to the surface of large particles		100		0.33		17.5, 61.5, 76.4, 135.8, 181.1, 429.4		3.3
aTEM range of particle diameters, agglomeration, shape, structure, electron beam sensitivity. bNanoproduct based on the Woodrow Wilson Nanotechnology Consumer Products Inventory (Woodrow Wilson International Center for Scholars 2011a).												

*Characterization of cosmetic powders in their original state.* Characterization of nanoparticles in the original products is necessary because the size distribution of particles released during a simulated product application might differ from that in the original product and may not adequately reflect the nanomaterial content to which a user would be exposed. We analyzed the powders in their original state using transmission electron microscopy (TEM) and laser diffraction spectrometry (LDS).

TEM. We used a transmission electron microscope (model 2010F; JEOL Ltd., Tokyo, Japan) to determine the size, shape, and agglomeration of electron-contrast particles [those visible in TEM photomicrographs; see Supplemental Material, p. 5 (http://dx.doi.org/10.1289/ehp.1104350)] in the tested powders. We spread small quantities of each product on HC300-Cu grids (Electron Microscopy Sciences, Hatfield, PA) and performed manual particle size measurement (Matyi et al. 1987) from the resulting photomicrographs using the automatically inserted scale marks.

Laser diffraction spectrometry. We used a laser diffraction particle size analyzer (Mastersizer 2000; Malvern Instruments Ltd., Worcestershire, UK) with a dry powder feeder (Scirocco 2000; Malvern Instruments Ltd.) to disperse the cosmetic powders in the air inside the device and determine their particle size distributions. The dry powder feeder employs a vibrating tray, which continuously feeds a powder into a Venturi tube, where it is accelerated close to the speed of sound. This separates loose agglomerates by shear forces (Jones 2002). Mastersizer 2000 uses the red helium neon laser (633 nm) to measure particle size from 2,000 μm down to 100 nm (Malvern Instruments Ltd. 2011). Additional information is provided in the Supplemental Material, p. 5 (http://dx.doi.org/10.1289/ehp.1104350).

The size distributions were generated by the Malvern Application (version 5.60) using the general purpose enhanced model for fine powders. All but one (i.e., regular powder E) of the products are mixtures of substances with different refractive indexes (RIs). The RI used in LDS is mathematically expressed as a complex number consisting of real and imaginary parts. The real part is the ratio of phase velocity of light in vacuum versus phase velocity of light in the bulk material, whereas the imaginary part, which describes absorption, depends on the nature and shape of particles (Gillespie and Lindberg 1992). Although the RIs of calcium carbonate, talc, and silica are around 1.5–1.7, the RI of titanium dioxide, a component of many cosmetic powders including one of the products tested in this study (nanopowder K), is 2.741. Because LDS performs analysis of a given powder based on a single RI, the accuracy of measurements may be undermined depending on the selected RI when particles with different RIs are present. To minimize the measurement error, and on the basis of the composition of the cosmetic powders [see Supplemental Material, Table 1 (http://dx.doi.org/10.1289/ehp.1104350)], we chose to perform our analyses using the RI of silica (1.544) for all powders with the exception of nanopowder K, for which we used the RI of zinc oxide (2.0041), which is a second active ingredient in this product along with titanium dioxide. This was considered a reasonable approach because the manufacturer did not provide the full composition of nanopowder K but listed only the active ingredients constituting 45% of the product: the nature of the remaining 55% of the product’s ingredients remained unknown. The imaginary RI could not be determined for the products experimentally, so we used the imaginary RI value of 0.1 as advised in the Malvern Application for ground transparent materials (Malvern Instruments Ltd.).

**Figure 1:**
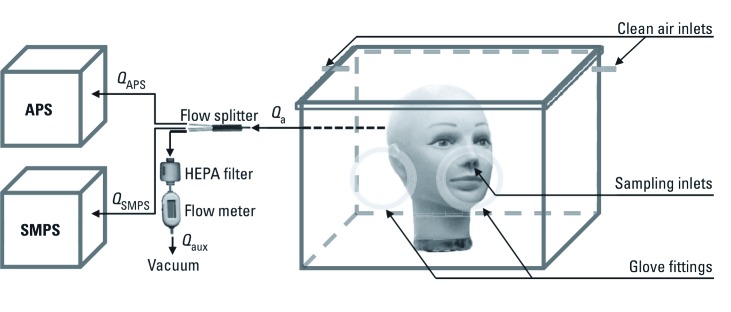
Experimental setup for simulated cosmetic powder application and measurement of resulting aerosol. *Q*_a_, total sampling flow rate; *Q*_APS_, aspiration rate of the APS; *Q*_aux_, auxiliary aspiration rate; *Q*_SMPS_, aspiration rate of the SMPS.

*Simulated application of cosmetic powders.* We realistically simulated the application of cosmetic powders and the resulting inhalation exposures using the experimental setup shown in Figure 1. We placed a human female mannequin head (Image Supply House, Endicott, NY) inside a custom-built glove box with a removable cover. The inner dimensions of the glove box were 56 × 33 × 39 cm^3^ (approximately 72 L), and we covered its inner walls with aluminum foil to reduce electrostatic effects. We placed the glove box inside a level 2 biosafety cabinet (NUAIRE Inc., Plymouth, MN) with inner dimensions of 178.4 cm wide × 71.8 cm high × 57.2 cm diameter. We removed the top cover of the glove box for 5 min immediately before each experiment, to bring the concentration of background particles to below the detection limit of the instruments used, and then replaced it. We operated the HEPA filtration system of the biosafety cabinet continuously throughout the powder application experiments. The glove box had two air inlets open to the inside of the biosafety cabinet to replace the air removed from the box by the measurement devices with particle-free air.

We applied all powders to the face of the mannequin head in a way that simulated actual product usage, using brushes or pads included with each product. Because the manufacturers did not include applicators with nanopowder M and regular powder E, we applied these two products using identical Kabuki brushes (Sephora USA Inc., San Francisco, CA). Additionally, we used a new Kabuki brush without any powder for comparison. After each application, we thoroughly cleaned the mannequin’s face with 70% vol denatured ethanol. We performed background (i.e., no manipulations in the glove box) control measurements between the product tests.

*Measurement of released particles.* We installed two stainless steel tubes with an inner diameter of 5 mm into the nostrils of the mannequin head to sample the particles that would be inhaled during the application of the powders. The two aerosol streams drawn through the mannequin’s nostrils were combined into one at the mannequin’s nape using a stainless steel Y-connector, fitted through the back wall of the glove box, and then split using a stainless steel flow splitter (model 3708; TSI Inc., Shoreview, MN) and drawn into a scanning mobility particle sizer (SMPS) (module combination 3080/3786; TSI Inc.) and an aersol particle sizer (APS; model 3321; TSI Inc.) via conductive tubing. These devices measured the actual airborne particle size distribution presented to the human respiratory system for inhalation, which is crucial for quantitative nanomaterial exposure assessment. The aspiration rate of the SMPS, *Q*_SMPS_, was 0.3 L/min, and that of the APS, *Q*_APS_, was 4.7 L/min. An additional pump provided an auxiliary aspiration rate, *Q*_aux_, of 6.0 L/min, thus resulting in the total sampling flow rate *Q*_a_ = 11.0 L/min, which corresponds to the breathing rate recommended for assessing short-term exposures for an 18- to 60-year-old female performing light activity (U.S. Environmental Protection Agency 1997) [see Supplemental Material, p. 5 (http://dx.doi.org/10.1289/ehp.1104350)]. All connectors and sampling lines were of conductive material, and as short and as vertical as possible, to minimize potential particle losses.

Because the SMPS provides a full scan of the entire size range in 3 min, we continuously and to the best of our ability uniformly applied each test powder during this time period. The APS measured particle concentration in all size bins simultaneously every second and provided an average concentration for each 3-min interval.

We used the SMPS system with a built-in 0.0457-cm impactor (*D*_50_ = 0.656 μm). This allowed us to obtain particle size distributions of 14.1–723 nm, whereas the APS measured particles in the 0.6–19.8 μm (600–19,800 nm) range. The measured particle size distributions by number are presented as Δ*N*/Δlog*D*_p_ per cubic centimeter, where Δ*N* is the number of particles detected in a size channel and Δlog*D*_p_ is the difference between the logarithms of the upper and lower channel diameters. The SMPS and APS measure electrical mobility and aerodynamic diameters, respectively, which are identical for spherical particles of 1 g/cm^3^ density. We assumed that all of the airborne powder particles had a density of 1 g/cm^3^, which we considered to be a reasonable approach because all investigated powders except regular powder E were composites of multiple materials, mixed in mostly unknown proportions. Therefore, we interpreted SMPS and APS data as measurements on the same particle-size scale.

We subtracted the background particle concentration data from each set of APS measurements. The SMPS measurements indicated particle concentrations below the detection limit in the overwhelming majority of size channels for the background and clean brush measurements. We tested each powder three times in randomized order and calculated the average particle-number–based size distributions for each powder and instrument.

## Results and Discussion

**Figure 2:**
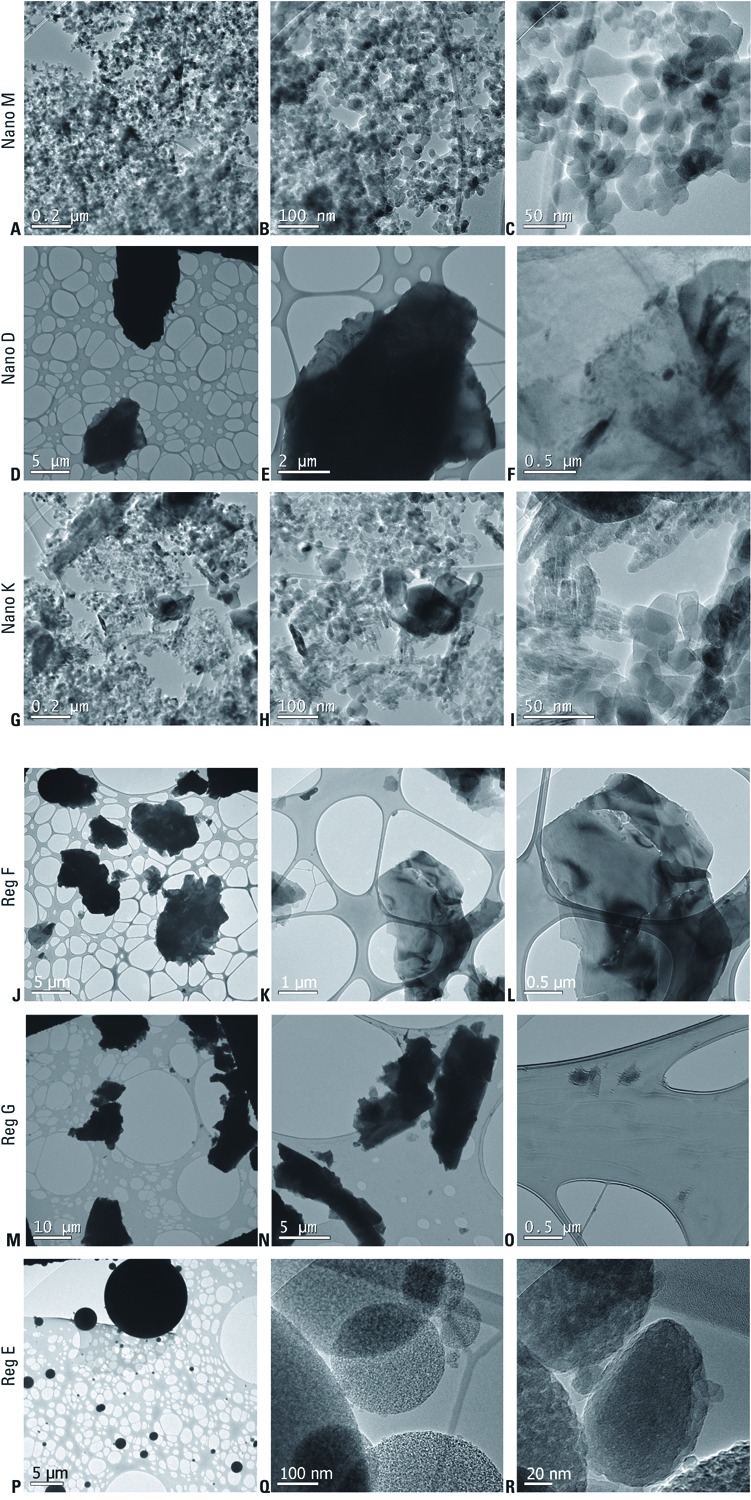
TEM photomicrographs of the tested cosmetic nanopowders (Nano) M (*A*–*C*), Nano D (*D*–*F*), and Nano K (*G*–*I*) and tested cosmetic regular powders (Reg) F (*J*–*L*), Reg G (*M*–*O*), and Reg E (*P*–*R*).

*Analysis of powders.* TEM analysis. TEM allows for direct viewing of solid electron-contrast primary nanoparticles or their agglomerates in consumer products. Representative TEM photomicrographs of tested powders are presented in Figure 2, and the summary of the TEM image analysis results is presented in Table 1. We found electron-contrast particles in all of the tested powders. The electron beam did not appear to alter the structure of any of the particles observed [see Supplemental Material, p. 6 (http://dx.doi.org/10.1289/ehp.1104350)].

All the primary particles (i.e., particles constituting the smallest dispersion level) in the sample of nanopowder M (Figure 2A–C) were in the nanosize range. In fact, the largest observed particle was 45 nm in diameter. No free nanoparticles or individual agglomerates were observed—the level of agglomeration was very high, because all the nanoparticles in the samples of this product were continuously interconnected on the TEM grids. The sample of nanopowder D (Figure 2D–F) contained no electron-contrast particles in the nanosize range. The only particles observed were > 5 μm (5,000 nm) in diameter and were not agglomerated. Nanopowder K (Figure 2G–I) contained a wide size range of highly agglomerated particles, with most primary particles being in the nanosize range. Close examination of photomicrographs for regular powder F (Figure 2J–L) showed nanosize particles in contact with larger particles. In the photomicrographs of regular powder G (Figure 2M–O), most of the surface of the TEM grid was covered with particles > 5 μm in diameter, with only a few separate nanoparticles. Regular powder E (Figure 2P–R) contained a large number of nanoparticles that were agglomerated and attached to larger particles.

Based on the composition of the powders as provided by the manufacturers [see Supplemental Material, Table 1 (http://dx.doi.org/10.1289/ehp.1104350)], we expect that the observed electron-contrast particles, including nanoparticles, contained silica (in all products with a possible exception of nanopowder K, for which information on composition was incomplete), talc (nanopowder D and regular powder G), mica (nanopowder D and regular powder F), aluminum hydroxide (nanopowder D), titanium dioxide and zinc oxide (nanopowder K), or kaolin and iron oxides (regular powders F and G).

Overall, based on TEM, we observed the highest abundance of nanoparticles in nanopowders M and K and in regular powder E.

**Figure 3:**
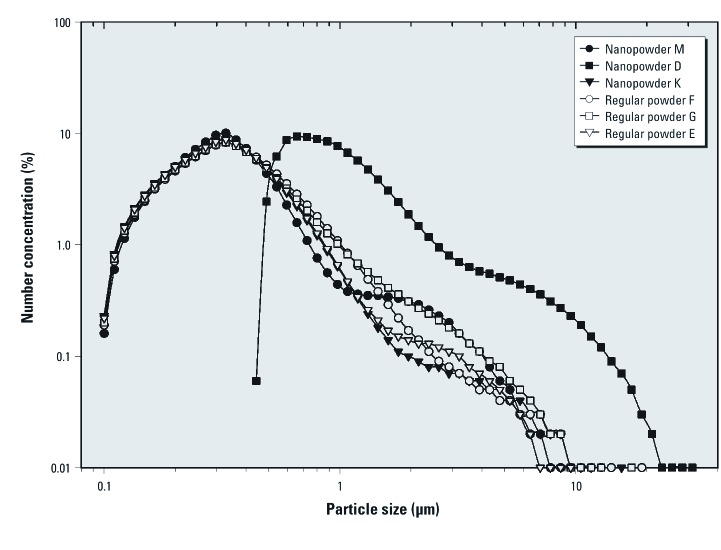
Size distributions of cosmetic powders by number as measured by the Mastersizer 2000. The data represent averages of three repeats.

LDS analysis. The summarized results of the LDS analysis are listed in Table 1. The instrument detected particles of 100 nm in nanopowders M and K and in regular powders F, G, and E. Size distributions of particles in these five powders were similar in shape, and all had mode diameters of 0.33 μm. Nanopowder D had a mode diameter of 0.66 μm (Figure 3).

Because the lower size limit of the LDS instrument used was 100 nm, particles with smaller diameter would not have been observed. However, the size distributions of these powders suggest that particles with diameters < 100 nm were likely present as well (Figure 3). This assumption is supported by the fact that TEM also registered particles < 100 nm in these five products.

Notably, neither TEM nor LDS indicated nanoparticles in nanopowder D, which is marketed as nanotechnology based. Conversely, the same analysis techniques detected a high number of nanoparticles in regular powder E, which is not marketed as nanotechnology based. These findings suggest that information provided regarding the presence or absence of nanomaterials in consumer products may not always be confirmed by experimental techniques.

**Figure 4:**
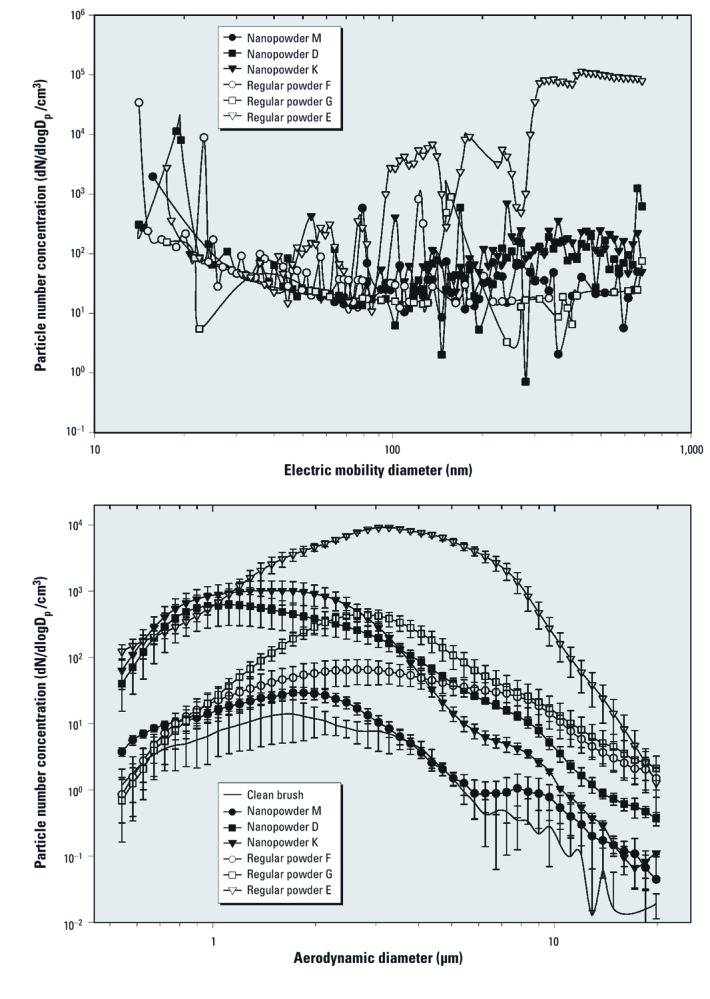
Size distributions of airborne cosmetic powders by number during their application to human mannequin face. The data represent averages of three repeats. (*A*) Electric mobility diameter measured by the SMPS: 14.1–723 nm measurement size range. (*B*) Aerodynamic diameter measured by the APS: 0.6–19.8 μm measurement size range. Error bars represent ± 1 SD.

*Analysis of airborne particles released during powder application.* The size distributions and concentrations of aerosol particles released during the simulated application of cosmetic powders based on SMPS and APS are presented in Figure 4. The mode diameters of the released particle size distributions that were sampled through the mannequin’s nostrils are provided in Table 1.

A detailed description of the particle size distributions is provided in Supplemental Material [see Supplemental Results, “Airborne Particle Measurement Results” (http://dx.doi.org/10.1289/ehp.1104350)]. In brief, for particles < 25 nm in diameter, which are characterized by higher alveolar deposition efficiency compared with larger particles (International Commission on Radiological Protection 1994), more variance in particle concentration was observed for nanopowders M and D and regular powders F and G (Figure 4A) than for the rest of the products. The SMPS system is very sensitive to fluctuating particle concentrations. Therefore, we concluded that for these four cosmetic powders (M, D, F, and G), airborne nanoparticle concentration in the region < 25 nm in diameter was unstable over the course of cosmetic powder application. In general, peak nanoparticle number concentrations for particles < 25 nm in diameter were comparable to the highest concentrations observed for particles that were 25–723 nm in diameter.

Concentrations of nanoparticles between 25 and 100 nm in diameter differed among products (Figure 4A). It is notable that the highest total particle counts were measured during the application of regular powder E, which is not marketed as a nanotechnology-based product by its manufacturer. Nevertheless, the spherical shape of the silica particles observed in this cosmetic powder using TEM (Figure 2B, P–R) suggests that they may have been engineered, which, if true, would make this product de facto nanotechnology based.

Airborne concentrations of particles between 100 nm and 20 μm in diameter (Figure 4) varied substantially among the different cosmetic powders. Particles across this entire range were measured during the application of both nanotechnology-based powders and regular powders, without obvious differences in the distributions between the nanopowders and regular powders. The products with the highest and lowest airborne concentrations varied within different particle size modes: fine (0.1–1 μm), accumulation (1–2.5 μm), coarse (2.5–10 μm), and supercoarse (> 10 μm), as defined by Lioy et al. (2006) (Figure 4B). Notably, for particle diameters > approximately 1.5 μm, regular power E had the highest concentrations and nanopowder M had the lowest concentrations [for additional details, see Supplemental Material, pp. 6–7 (http://dx.doi.org/10.1289/ehp.1104350)].

It is important to note, however, that application of all nanopowders resulted in the release of particles as large as 20 μm (Figure 4B), and judging from the size distribution, even larger particles may have been released. As shown by the electron microscopy (Figure 2), the nanoparticles were agglomerated in the cosmetic powders, which suggests that nanomaterial may have been present in all airborne particle size fractions generated in the personal breathing cloud by cosmetic powder application.

SMPS (Figure 4A) and APS (Figure 4B) measurements in the overlapping size range (500–700 nm or 0.5–0.7 μm) do not always agree. A discussion of potential causes for such differences is provided elsewhere (Nazarenko et al. 2011).

*Implications for exposure assessment and health risks.* Although deposition in the alveolar region of the lung is the highest for nanoparticles and agglomerates of nanoparticles < 100 nm in diameter, particles larger than approximately 0.3 μm (300 nm) in diameter can efficiently deposit in the non–gas-exchange region of the lung, with particles > 10 μm in diameter (supercoarse particles) depositing primarily in the head airways (Hinds 1999). Therefore, inhalation of aerosol particles containing nanomaterials in both the 1–100 nm and 100 nm to 20 μm diameter size ranges, and possibly larger, and their potential deposition in all regions of the respiratory system, should be considered.

Our TEM data showed a predominance of agglomerated nanoparticles in nanopowders M and K, and a high number of agglomerated nanoparticles that were in contact with the surface of larger particles in regular powder E. Based on the TEM and aerosol measurement data, we expect that most of the airborne nanomaterial from cosmetic powders, especially by mass [see Supplemental Material, Figure 1 (http://dx.doi.org/10.1289/ehp.1104350)], will be in agglomerated form in particle size fractions > 100 nm, which are usually not the focus of most toxicology studies involving nanomaterials. A similar phenomenon could be predicted for many other nanotechnology-based consumer products that release nanoparticles as agglomerates and/or as composites with larger particles.

Most toxicological studies of potential health effects of inhaled nanoparticles, including studies of murine models, have used aerosols in which individual nanoparticles or nanosize agglomerates are a dominant fraction. For example, Geiser et al. (2005) administered a pure conditioned titanium dioxide aerosol with a 22-nm count median diameter into the rat respiratory system through an endotracheal tube. Sayes et al. (2010) used a freshly generated silica aerosol with 37- and 83-nm mode diameter aerosols for nose inhalation exposure of rats. Based on size, such particles would primarily deposit in deep regions of the respiratory system.

By contrast, in consumer products such as cosmetic powders, our findings suggest that primary particles would likely coagulate among themselves and with other ingredients present in the product before its application. As a result, aerosols produced when cosmetic powders are used may be dominated by much larger particles, including agglomerates ≥ 10 μm in diameter. Consequently, application of cosmetic powders may result in inhaled nanomaterial deposition not only in the gas-exchange region of the lung (alveoli) but also in the non–gas-exchange regions (tracheobronchial and head airways). For example, nanoparticles ≤ 100 nm may form agglomerates > 10 μm (supercoarse-size particles) that deposit much higher up in the respiratory system than do nonagglomerated nanoparticles, that is, in the head airways rather than the alveolar and tracheobronchial regions (International Commission on Radiological Protection 1994), resulting in completely different health effects. Use of pure nanomaterials, as in the experimental studies cited above, would lead to a much higher nanomaterial deposition in the deeper regions of the respiratory system than could be expected based on product exposure simulation. As a result, such studies would have a diminished capacity to predict human health effects due to exposure to actual nanotechnology-based products.

The combined surface area of nanoparticle agglomerates exceeds that of solid particles of the same size by orders of magnitude. Thus, such agglomerates would present a much higher potential for surface-based reactivity within live tissue, potentially leading to greater health risks compared with solid particles of the same size (Brown et al. 2001; Duffin et al. 2007; Gwinn and Vallyathan 2006; Monteiller et al. 2007; Nel et al. 2006).

At the same time, depending on the breathing rate, the laryngeal jet may break up inhaled loose agglomerates as small as 1 μm in diameter (Li et al. 1996) into smaller aggregates or individual particles that could deposit throughout the entire respiratory system. Therefore, quantitative nanoparticle exposure studies should take into account the polydisperse nature of aerosol produced during the use of nanotechnology-based consumer products and examine not only the exposure to and deposition of unbound nanoparticles, but also the fate, transport, and deposition of nanoparticle agglomerates in all regions of the respiratory system, including smaller aggregates or particles that may result from the breakup of larger nanoparticle agglomerates.

Our findings on potential nanomaterial inhalation exposure due to the use of actual consumer products emphasize that properties and effects of the pure nanomaterial ingredients cannot be used to predict actual consumer exposures and resulting health effects. Therefore, experimental techniques for toxicity studies of de facto nanotechnology-based consumer products must be developed. Results of such studies will provide guidance for the developing market of nanotechnology-based consumer products and help clarify the need and feasibility of its regulation.

We performed our measurements indoors at comfortable relative humidity levels: 40–50%. Application of the powders at different humidity conditions, especially at very low or very high levels, could possibly affect the extent of powder agglomeration and thus its deposition in the respiratory system. The effect of relative humidity and other environmental conditions on the extent of exposures should be addressed in future studies.

## Conclusions

The release of particles > 100 nm and as large as 20 μm in diameter indicates potential exposure to nanoparticle agglomerates, especially from products in which a very large proportion of primary particles are in the nanosize range (e.g., nanopowders M and K and regular powder E, as shown by the TEM).

TEM observations and aerosol measurements suggest that exposure to nanomaterial(s) due to the use of cosmetic powders will be predominantly in the form of agglomerates or nanomaterials attached to larger particles that would deposit in the upper airways of the human respiratory system rather than in the alveolar and tracheobronchial regions of the lung, as would be expected based on the size of the primary nanoparticles. This may lead to completely different health effects than expected on the basis of nanoparticle behavior and toxicology studies for the alveolar region. Thus, predominant deposition of nanomaterials in the upper airways must be taken into account when designing inhalation toxicology studies of actual consumer products.

We conclude that the use of de facto nanotechnology-based cosmetic powders has a strong potential to result in inhalation exposure to single and agglomerated nanoparticles and that this potential should be quantitatively described by exposure assessment studies.

The absence of information regarding the engineered status of nanomaterials in consumer products and difficulties in determining whether engineered nanomaterials are present point to the need for legislation requiring manufacturers to report the use of engineered nanomaterials in their products. It is also important to determine whether nanosize particles present in some consumer products may be the result of a manufacturing process rather than a consequence of the deliberate introduction of engineered nanomaterial(s) into the products.

This study provides an example of data acquisition methodology that could be used in quantitative exposure studies of nanotechnology-based consumer products, especially when simulating realistic exposure scenarios. Results from such studies could be used to estimate exposures resulting from the short-term and long-term use of cosmetic powders and to design future studies of nanoparticle deposition in the respiratory system and inhalation toxicology experiments.

## Supplemental Material

(262 KB) PDF
