# Supplementary material for: Potential for Inhalation Exposure to Engineered Nanoparticles from Nanotechnology-Based Cosmetic Powders
Source: Environ Health Perspect. 2012 Mar 6;120(6):885–92. doi: 10.1289/ehp.1104350 (PMC3385434; doi:10.1289/ehp.1104350)
Supplement: (262 KB) PDF [file ehp.1104350.s001.pdf]

Supplemental Material

Potential for inhalation exposure to engineered nanoparticles from nanotechnology-based cosmetic powders

Yevgen Nazarenko[1], Huajun Zhen[1], Taewon Han[1], Paul J. Lioy[2,3], and Gediminas Mainelis[1,3,*]

[1] Rutgers University, Department of Environmental Sciences, New Brunswick, NJ 08901;
[2] RWJMS-UMDNJ, Piscataway, NJ 08854;
[3] Environmental and Occupational Health Sciences Institute sponsored by Robert Wood Johnson Medical School (RWJMS)-UMDNJ and Rutgers University, Piscataway, NJ 08854.

This pdf file includes:

Supplemental Material, Table 1 2
Supplemental Material, Table 2 and Table 3 3
Supplemental Material, Figure 1 4
Supplemental Notes 5
Supplemental Results 6
References 8

Supplemental Material, Table 1. Tested cosmetic powders

Product	Composition[b]	Purpose[b]
Nanopowder M[a]	Water, Butylene glycol, Sodium ascorbyl phosphate, Glycerin, Betain, Silica, Dimethicone, Citric acid, Polymethyl metacrylate, Squalane, Sodium hydroxide, Sodium metabisulfite, Capryloyl glycine, Sodium Hyaluronate, Marus Alba root extract, Rosmarinus Officinalis (Rosemary) leaf extract, olea europaea (Olive) leaf extract	Powder Moisturizer
Nanopowder D[a]	Mica, Talc, Dimethicone/Vinyl Dimethicone crosspolymer, Hydrogenated C6-14 Olefin polymers, Petrolatum, Dimethicone, Polysilicone-2, Aluminum stearate, HDI/Trimethylol Hexyllactone crosspolymer, Sorbitan sesquisostearate, Aluminum hydroxide, Methicone, Tocopherol, Silica, Triisostearin, Trimethylolpropane trioctanoate, Ethylparaben, Butylparaben, Parfum, CI 77492, CI 77947, CI 77891, CI 77491, CI 77499	Powder Blusher
Nanopowder K[a]	Active Ingredients: Titanium dioxide – 25%, Zinc Oxide – 20%	Powder Sunscreen
Regular Powder F	Dimethicone, Silica, Kaolin, Water, Hydrolyzed Soy Protein, Caprylyl glycol, Hexylene glycol, Methicone, Coconut acid, Phenoxyethanol, +/- Mica, Iron oxides (CI 77491, CI 77492, CI 77499), ILN31255	Blot Powder
Regular Powder G	Talc, C12-15 Alkyl Benzoate, Kaolin, Silica Silylate, +/- Mica, Iron oxides (CI 77491, CI 77492, CI 77499)	Blot Powder
Regular Powder E	Silica	Finishing Powder

[a]Nanoproduct as per the Woodrow Wilson Nanotechnology Consumer Products Inventory
[b]As per manufacturer

Supplemental Material, Table 2. Descriptive statistics of the size distributions of cosmetic powders by number as measured by the Mastersizer 2000. These size distributions are shown in Figure 3 (main text).

	Nanopowders			Regular Powders		
	M	D	K	F	G	E
Mode, µm	0.33	0.66	0.33	0.33	0.33	0.33
Geometric Mean (d_g), µm	0.33	1.03	0.32	0.35	0.35	0.33
Geometric Standard Deviation (σ_g)	1.76	1.83	1.72	1.79	1.86	1.73

Supplemental Material, Table 3. Descriptive statistics of the size distributions of cosmetic powders by number during their application to human mannequin face as measured by the Aerodynamic Particle Sizer (APS). These size distributions are shown in Figure 5 (main text).

		Nanopowders			Regular Powders		
	Clean Brush	M	D	K	F	G	E
Mode, µm	1.72	1.72	1.04	1.49	2.64	2.64	3.28
Geometric Mean (d_g), µm	1.75	1.64	1.44	1.45	2.86	2.79	3.12
Geometric Standard Deviation (σ_g)	1.70	1.69	1.66	1.54	1.93	1.56	1.63

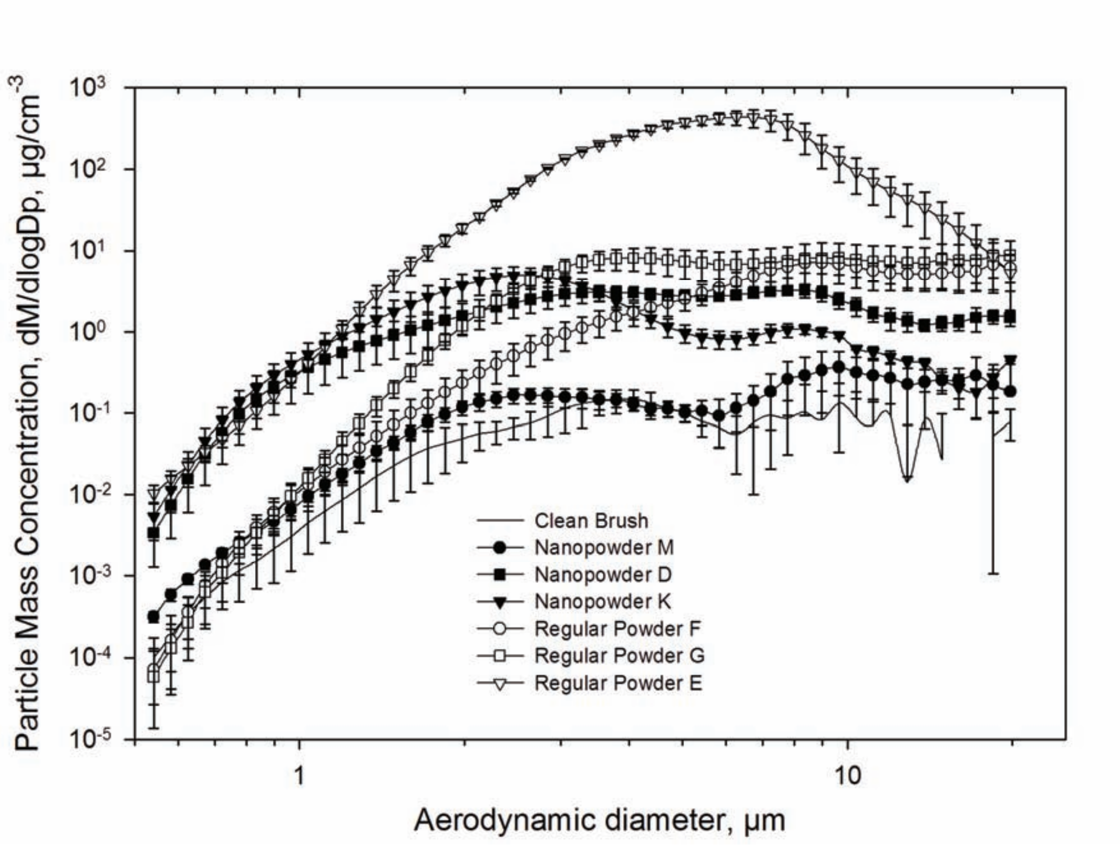

Supplemental Material, Fig. 1. Size distributions of airborne cosmetic powders by mass during their
application to human mannequin face as measured by the Aerodynamic
Particle Sizer (APS): 0.6 - 19.8 μm measurement size range.
The data represent averages of three repeats with error bars representing
± one standard deviation based on these repeats.
Nanotechnology-based cosmetic powders are shown in black symbols, regular
ones are shown in white symbols.

Supplemental Notes

Material Visibility with TEM

Only certain types of nanoparticles, e.g. certain metal, metal oxide, other inorganic and some organic nanoparticles absorb and scatter electrons enough to be visible in TEM micrographs (Egerton et al. 2004). Weak phase objects (mostly organic material) have low electron contrast and are consequently not visible in TEM images.

Mastersizer 2000's Operation

The laser light undergoes scattering, diffraction, and absorption by the airborne material, which results in varying intensities of the signal measured by large angle, focal plane, and backscatter detectors (Malvern Instruments Ltd 2011). The size, shape, and nature of the particles determine light scattering through reflection and refraction. The light resulting from diffraction depends solely on the geometric cross-section of the particle. Absorption is determined by the size and nature of the particles (Hackley et al. 2004). Mie theory is applied to determine particle size distribution.

The primary measurement unit is particle volume concentration. The instrument's software converts volume based scattering data into a particle size frequency distribution. For non-spherical particles, their size is reported as volume-equivalent diameter of a sphere.

Choice of the Inhalation Flow Rate

The U.S. EPA 1997 Exposure Factors Handbook (Table 5-14) recommends the used inhalation flow rate specifically for short- term exposures for our chosen user/activity profile. We believe that our choice of the recommended inhalation flow rate for short-term exposures matches the type of inhalation exposure expected during cosmetic powder application (short-term exposure) and is the most realistic relative to the activity level expected during cosmetic powder application. This inhalation flow rate slightly exceeds the inhalation flow rates referenced for sedentary activity defined as sitting and standing (Table 5-6) and as car driving and riding (Table 5-7). We find it consistent with our referenced inhalation flow rate since application of a cosmetic powder would occur during both sitting or standing, but performing the physical activity required for the application of a product and the same application but during a visit to a public bathroom or a similar place of retreat where a cosmetic powder application process would follow physical movement that would be more intense than simply standing or sitting, which would result in a somewhat higher inhalation flow rate.

Electron Beam Sensitivity

When material is irradiated in TEM above a certain magnification setting (Carlo et al. 2002; Leapman and Sun 1995 ; Turgis and Coqueret 1999), higher electron beam power density per unit area of the sample results in physical and/or chemical alteration of the tested material (Egerton et al. 2004; Hobbs 1987). During the TEM analysis, this process can be observed visually. As mostly organic nanoparticles tend to be beam sensitive (Egerton et al. 2004), it can be concluded with some degree of certainty about organic or inorganic nature of nanoparticles in the tested products based on beam sensitivity.

Supplemental Results

Airborne Particle Measurement Results

The particle concentrations for 14.1 – 700 nm size range as measured by the SMPS are shown in Figure 4 (main text). In the nanosize range (14.1 nm – 98.2 nm), the highest concentration reached 3.4×10^{4} cm^{-3} (at 14.1 nm for Regular Powder F). Below 25 nm, Nanopowders M and D and regular Powders F and E showed spikes of high nanoparticle concentration. The instability of the aerosol concentration over the course of cosmetic powder application to the face of the mannequin mimics the real life situation and is not unexpected. The impact of this instability on the results is discussed in the main article.

In the rest of the nanosize range (25 – 98.2 nm), Regular Powder G remained comparatively low reaching only 2.4×10^{1} cm^{-3} (at 53.3 nm) while Regular Powder E consistently showed the highest concentrations among the investigated powders with three maxima at 61.5, 76.4, and 98.2 nm (3.1×10^{2}, 3.6×10^{2}, and 2.8×10^{3} cm^{-3} respectively).

From ~100 nm to ~700 nm, concentration of Regular Powder E was the highest reaching the order of 10^{5} cm^{-3} for ~300 – 700 nm particles. Concentrations of the rest of the powders ranged from 7.2×10^{-1} cm^{-3} (at 278.8 nm) to 1.3×10^{3} cm^{-3} (at 661.2 nm) both for Nanopowder D. The background SMPS measurement and the clean brush control showed concentrations mostly below the detection limit of the instrument and are therefore not shown in Figure 4 (main text).

Results for 0.6-20 µm particles as measured by the APS are shown in Figure 5 (main text). In the size range from 0.6 to 1 µm, the lowest concentrations were observed during application of Regular Powders F and G, and Nanopowder M with concentrations reaching ~10^{1} cm^{-3}, while the other three powders reached concentrations up to 10^{3} cm^{-3}.

The concentration of Nanopowder M was the lowest for the rest of the size range and comparable to the level of the clean brush control.

In accumulation mode (1 – 2.5 μm), moderate concentrations of particles were released during application of Powders F and G reaching only 6.5×10^1 and 4.2×10^2 cm^{-3} at 2.5 μm. The highest concentration in this range was from Regular Powder E reaching close to 10^4 cm^{-3}. For the Nanopowders D and K the concentrations were approximately 10^3 cm^{-3}.

In the coarse (2.5 – 10 μm) and supercoarse (>10 μm) size modes, the highest concentrations were observed from Regular Powder E: it peaked at 7.8×10^3 cm^{-3} at 3 μm and decreased to approximately 1.3×10^1 cm^{-3} in the supercoarse mode. The particle concentration from nanopowders D and K and regular powders F and G were substantially higher than for Nanopowder M. At 2.5 μm size, their concentrations ranged from 6.9×10^1 cm^{-3} to 5.1×10^2 cm^{-3}. For larger particles, concentrations of these powders declined and separated a little bit more. At 10 μm, concentrations of these four powders ranged from 7.8×10^{-1} cm^{-3} to 1.1×10^1 cm^{-3}.

References

Carlo SD, El-Bez C, Alvarez-Rua C, Borge J, Dubochet J. 2002. Cryo-negative staining reduces electron-beam sensitivity of vitrified biological particles. J Struct Biol 138: 216-226.

Egerton RF, Li P, Malac M. 2004. Radiation damage in the TEM and SEM. Micron 35(6): 399-409.

Hackley VA, Lum L, Gintautas V, Ferraris CF. 2004. Particle Size Analysis by Laser Diffraction Spectrometry: Application to Cementitious Powders. NISTIR 7097.Technology Administration, U.S. Department of Commerce.

Hobbs LW. 1987. Electron-beam sensitivity in inorganic specimens. Ultramicroscopy 23(3-4): 339-344.

Leapman RD, Sun S. 1995 Cryo-electron energy loss spectroscopy: observations on vitrified hydrated specimens and radiation damage. Ultramicroscopy 59: 71-79.

Malvern Instruments Ltd. 2011. Laser Diffraction Particle Sizing. Available: http://www.malvern.com/LabEng/technology/laser_diffraction/particle_sizing.htm [accessed 11 January 2011].

Turgis J-D, Coqueret X. 1999. Electron beam sensitivity of butyl acrylate copolymers: effects of composition on reactivity. Macromol Chem Phys 200: 652–660.
